# Thoracobifemoral bypass for infrarenal aortic occlusion caused by retroperitoneal fibrosis

**DOI:** 10.1016/j.jvscit.2022.01.005

**Published:** 2022-01-26

**Authors:** Kathy K. Wang, Rym El Khoury, Axel Joob, Chad E. Jacobs, John V. White, Lewis B. Schwartz

**Affiliations:** a Department of Surgery, Advocate Lutheran General Hospital, Park Ridge, Ill; b Division of Vascular Surgery, University of California, San Francisco, Calif

**Keywords:** Thoracobifemoral bypass, Aortic occlusion, Retroperitoneal fibrosis

## Abstract

Retroperitoneal fibrosis (RPF) is an uncommon fibrotic disorder that can cause pain, ureteral obstruction, deep venous thrombosis, hydrocele, and, rarely, aortic occlusion. Herein is described a 65-year-old man with aortic occlusion from idiopathic RPF who was treated with axillobifemoral bypass grafting, which failed in the intermediate term. On representation with critical claudication, he underwent thoracobifemoral bypass grafting via a lateral retroperitoneal tunnel created through a midline, infraumbilical counterincision. He was discharged home on postoperative day 5. This illustrates the successful use of thoracic aortic inflow to treat the aortoiliac occlusive complication of RPF.

Retroperitoneal fibrosis (RPF) was first recognized in 1948 by the urologist John Ormond,^1^ who described a fibrotic process that entraps the ureters. RPF is increasingly recognized but remains rare, with estimated yearly incidence 0.1 to 1.3/100,000 people.^2^ Although most cases are idiopathic, the condition may also be caused by malignancy, prior surgery, radiotherapy, infection, connective tissue disorder, medication reactions, and/or inflammatory aneurysm.^3^

The most frequent complication of RPF is renal failure secondary to ureteral obstruction, but vascular compression of major vessels also occurs.^2^, ^3^, ^4^, ^5^, ^6^, ^7^, ^8^ Aortic occlusion from RPF is decidedly rare.^6^^,^^8^^,^^9^ When diagnosed, direct aortic reconstruction is ill-advised due to the lack of defined tissue planes. Treatment with endovascular methods or extra-anatomic bypass has been applied with good results.^10^^,^^11^

Herein, we report a patient who previously underwent axillobifemoral bypass grafting for RPF-induced aortic occlusion, but developed recurrent claudication and graft thrombosis. He was successfully revascularized with thoracobifemoral bypass, with the graft tunneled in a lateral retroperitoneal space created through an infraumbilical counterincision. The patient provided informed consent for publication.

## Case report

A 65-year-old gentleman with hypertension, dyslipidemia, coronary artery disease, type 2 diabetes, history of right popliteal deep venous thrombosis on warfarin, and a 35 pack-year smoking history was diagnosed with idiopathic RPF 2 years prior in another state. He had periaortic thickening causing left renal artery, infrarenal aortic, and common iliac occlusion. The patient initially underwent axillobifemoral bypass grafting but developed graft thrombosis 2 years later. During an attempt at revision, significant scarring of the groin and axilla was encountered, so the procedure was terminated without attempt at restoring flow.

Two months later, he presented to our institution for evaluation. He reported significant claudication after walking approximately 50 feet, which prevented meaningful ambulation and employment. On physical examination, he had no pulsations in the subcutaneous graft limbs. Femoral and pedal pulses were absent. His feet were cool but without lesions or gangrene.

**Fig 1:**
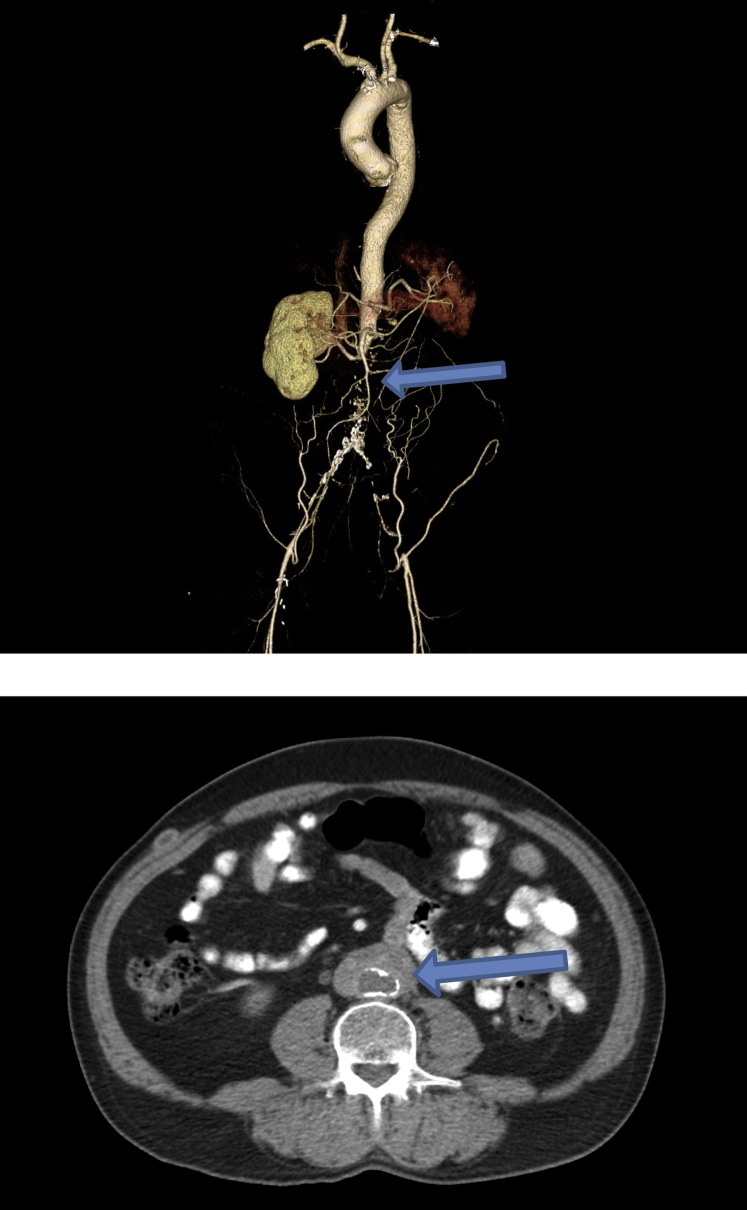
Computed tomographic angiography (CTA) in a 65-year-old man with retroperitoneal fibrosis (RBF) and aortic occlusion. The top panel shows a 3D reconstruction of the aortoiliac occlusion (*arrow*); note the absence of dye in the axillobifemoral bypass graft, which is also occluded. The bottom panel shows the occluded infrarenal aorta in cross-section incorporated within a dense, periaortic mass typical of RBF (*arrow*).

Computed tomography angiography (CTA) revealed a normal-appearing thoracic aorta, occlusion of the axillobifemoral bypass graft, and an amorphous retroperitoneal mass surrounding the occluded infrarenal aorta, common iliac, internal iliac, and external arteries (Fig 1). There was chronic periaortitis, left renal artery occlusion with left kidney atrophy, extensive pelvic collateralization with reconstitution of the distal bilateral external iliac arteries, and patency of the common femoral arteries.

A thoracobifemoral bypass was recommended. The patient was taken to the operating room and placed in the modified right lateral decubitus position, with the left chest rotated and elevated 30°, the left arm supported above the head, and the hips kept flat to facilitate bilateral groin exposure. General single-lung orotracheal anesthesia was induced. A longitudinal incision was made in the right groin and the dissection carried down to the prior occluded graft, with its distal anastomosis at the right femoral bifurcation. Likewise, the occluded left graft was identified with its distal anastomosis at the left femoral bifurcation. Bilateral superficial femoral and profunda femoris arteries were patent and soft.

A 10-cm lower midline incision was made below the umbilicus to expose the retroperitoneum. The rectus fascia was incised and the musculature retracted to the right. The transversalis fascia was incised and the abdominal contents were retracted to the right, exposing the left retroperitoneum. Blunt dissection was used to create retroperitoneal tunnels to each of the groin incisions. No fibrotic mass in the left lateral retroperitoneum was encountered.

A left lateral thoracotomy through the eighth interspace was made with shingling of the eighth rib. The inferior pulmonary ligament was excised to expose the thoracic aorta, which was soft and normal in caliber. Blunt dissection was used to create a tunnel in the lateral left diaphragm, bridging the retroperitoneal and thoracic incisions.

A total of 5000 units of intravenous heparin were administered. A 20 mm × 10 mm bifurcated Dacron graft was delivered to the field, and the proximal anastomosis to the thoracic aorta completed end-to-side. The left limb of the graft was delivered through the retroperitoneal tunnel to the left groin. To extend the right limb of the graft to reach the right groin, a straight segment of 10-mm Dacron was sutured end-to-end to and was delivered through the retroperitoneum. The anastomoses were completed in a standard fashion to the bilateral superficial femoral arteries. The prior thrombosed graft was not removed. An anterior chest tube was inserted.

**Fig 2:**
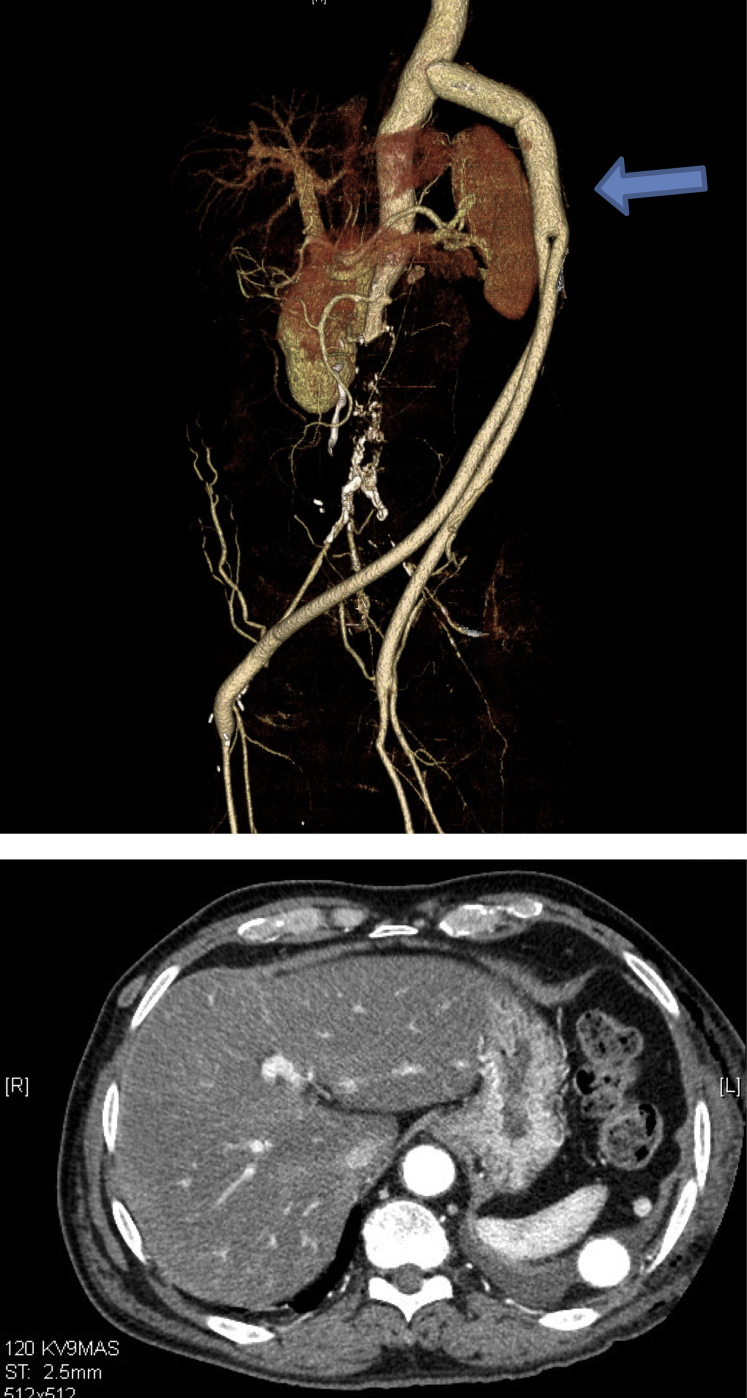
Computed tomographic angiography after thoracobifemoral bypass grafting including vascular reconstruction (top) and cross-section (bottom). Note the tunnel of the main body of the graft in the left lateral diaphragm (*arrow*).

The patient was extubated in the operating room. The operative time was 4 hours 13 minutes, and estimated blood loss was 300 mL. His recovery was uncomplicated. The chest tube was removed on the third postoperative day, and he was discharged home on the fifth postoperative day. Repeat venous duplex before discharge showed persistent right popliteal deep venous thrombosis with extension to the right femoral vein; CTA chest did not demonstrate pulmonary embolus. He was discharged on full-dose enoxaparin with transition to warfarin and started on aspirin 81 mg daily. His symptoms resolved, and a follow-up CTA at 1 month showed good flow through the widely patent graft (Fig 2). The patient remained with a patent graft at 4-month follow-up.

## Discussion

Use of the thoracic aorta as inflow for aortobifemoral bypass was introduced in 1961^12^^,^^13^ and popularized over the ensuing decades.^14^, ^15^, ^16^, ^17^, ^18^, ^19^, ^20^, ^21^, ^22^, ^23^ The procedure was originally indicated for patients with critical limb ischemia in the presence of a hostile abdomen from prior surgery, irradiation, or infection. Although technically demanding, the operation proved durable with extended long-term patency and acceptable operative morbidity.^17^^,^^18^ The operation is maximally invasive, but advantages of the avoidance of celiotomy, the provision of inflow from a large and normal aorta, and the directness of revascularization are attractive. Although performed infrequently in the modern era of vascular intervention, the operation remains effective, with recent studies showing 1-year primary patency over 85% and 1-year survival over 90%.^24^, ^25^, ^26^

Herein is reported the use of this historic procedure for aortic occlusion as a complication of RPF. Given severe pathology and complex occlusion demonstrated on imaging, endovascular reconstruction was not considered. It was felt that endovascular intervention carried a prohibitive risk of damage to the right renal artery, given that the aorta was occluded immediately distal to its origin, and the process had already destroyed the left renal artery. There was no landing zone in the perirenal aorta. The thoracobifemoral approach was selected in hopes of providing sufficient inflow, avoiding potentially fibrotic fields, and achieving a durable result.

Much has been written about the unusual and anatomically complex tunnel that must be created for thoracobifemoral bypass grafting.^14^^,^^15^^,^^21^^,^^27^^,^^28^ The Dacron graft is typically routed bluntly through an incision in the paravertebral posterior diaphragm, in the left retroperitoneum posterior to the kidney, lateral to the left iliac artery on the surface of the left psoas, and then under the left inguinal ligament for anastomosis to the left common femoral artery. The right leg is typically revascularized via femorofemoral bypass.^14^^,^^15^^,^^21^^,^^27^ Here, we tunneled the graft under direct vision using a counterincision that exposed the left lateral retroperitoneum. This enabled the use of a bifurcated graft with its right limb tunneled to the right groin, permitting advantages of optimized flow dynamics in a deep-seated, nonsubcutaneous location.

## Conclusions

The use of thoracobifemoral bypass to treat aortic occlusive disease can be a successful and durable remedial procedure for failed extra-anatomical bypass, and should especially be considered when there is intra-abdominal pathology to be avoided.
